# Baseline Functional Connectivity Features of Neural Network Nodes Can Predict Improvement After Sound Therapy Through Adjusted Narrow Band Noise in Tinnitus Patients

**DOI:** 10.3389/fnins.2019.00614

**Published:** 2019-07-04

**Authors:** Lv Han, Zeng Na, Liu Chunli, Chen Yuchen, Zhao Pengfei, Wang Hao, Cheng Xu, Zhang Peng, Wang Zheng, Yang Zhenghan, Gong Shusheng, Wang Zhenchang

**Affiliations:** ^1^Department of Radiology, Beijing Friendship Hospital, Capital Medical University, Beijing, China; ^2^National Clinical Research Center for Digestive Diseases, Beijing Friendship Hospital, Capital Medical University, Beijing, China; ^3^Department of Otolaryngology Head and Neck Surgery, Beijing Friendship Hospital, Capital Medical University, Beijing, China; ^4^Department of Otolaryngology, Affiliated Hospital of Chengde Medical College, Chengde, China; ^5^Department of Radiology, Nanjing First Hospital, Nanjing Medical University, Nanjing, China

**Keywords:** tinnitus, sound therapy, functional magnetic resonance imaging, functional connectivity, degree centrality, neural biomarker, graph theory

## Abstract

Previous resting-state functional magnetic resonance imaging (fMRI) studies have shown neural connectivity alterations after the treatment of tinnitus. We aim to study the value of the baseline functional connectivity features of neural network nodes to predict outcomes of sound therapy through adjusted narrow band noise. The fMRI data of 27 untreated tinnitus patients and 27 matched healthy controls were analyzed. We calculated the graph-theoretical metric degree centrality (DC) to characterize the functional connectivity of the neural network nodes. Therapeutic outcomes are determined by the changes in the Tinnitus Handicap Inventory (THI) score after a 12-week intervention. The connectivity of 10 brain nodes in tinnitus patients was significantly increased at baseline. The functional connectivity of right insula, inferior parietal lobule (IPL), bilateral thalami, and left middle temporal gyrus was significantly modified with the sound therapy, and such changes correlated with THI changes in tinnitus patients. Receiver operating characteristic curve analyses revealed that the measurements from the five brain regions were effective at classifying improvement after therapy. After age, gender, and education correction, the adjusted area under the curve (AUC) values for the bilateral thalami were the highest (left, 0.745; right, 0.708). Our study further supported the involvement of the fronto-parietal-cingulate network in tinnitus and found that the connectivity of the thalamus at baseline is an object neuroimaging-based indicator to predict clinical outcome of sound therapy through adjusted narrow band noise.

## Introduction

Tinnitus, the perception of sound without any external stimuli, is highly prevalent, affecting 10 to 25% of the general population (Baguley et al., 2013; Bauer, 2018). Without effective treatment, patients with persistent tinnitus experience substantial distress that significantly affects their quality of life, even leading to suicide. New and effective therapeutic approaches are desperately needed.

Current therapeutic approaches include tinnitus retraining therapy (Scherer et al., 2014; Kim et al., 2016), counseling, cognitive behavior therapy (Conrad et al., 2015; Weise et al., 2016), transcranial magnetic stimulation (Kreuzer et al., 2017; Formanek et al., 2018), hearing aids (Searchfield et al., 2010), pharmacological treatments (von Boetticher, 2011), and several innovative sound-based treatments (e.g., Heidelberg Neuro-Music Therapy) (Krick et al., 2015, 2017a,b), Cochleural Alternating Acoustic Beam Therapy (CAABT) (Liu et al., 2018), and Tailor-Made Notched Music Training (TMNMT) (Okamoto et al., 2010; Kim et al., 2017; Lee et al., 2017). Sound therapy is considered as a cost-effective management for tinnitus (Tunkel et al., 2014; Walker et al., 2016; Makar et al., 2017) and is one of the most commonly used first-line therapy interventions for decades (Henry et al., 2002). An adjusted narrow band noise was commonly used in sound therapy. For tinnitus patients, sound therapy may not totally eliminate the symptoms, but it could help patients become familiar with tinnitus and offer relief from tinnitus-related distress, subsequently improving their quality of life (Hobson et al., 2007, 2012; Newman and Sandridge, 2012; Hoare et al., 2014; Aytac et al., 2017; Mahboubi et al., 2017). Determining the factors that can predict the treatment efficacy of sound therapy will help to deliver cost-effectiveness therapy in tinnitus patients.

For patients with tinnitus, the functional connectivity differs from that of control individuals (Husain, 2016). Effective treatments for tinnitus have been shown to correlate with alterations in brain activities. Brain regions in the posterior cingulate cortex (PCC)/precuneus (Krick et al., 2017a), rostral and pregenual anterior cingulate cortices (rACC/pgACC), auditory cortex, parahippocampus (Kim et al., 2016), and inferior frontal gyrus (Roland et al., 2015) were reported to have significantly altered functional activity after therapy. However, very few studies have investigated the value of a non-invasive test, i.e., neuroimaging to predict clinical outcome, especially with sound therapy through adjusted narrow band noise. If a set of parameters is determined through such study, it would be valuable to guide patient selection and to deliver personalized therapeutic strategies.

Among numerous neural network measures, degree centrality (DC) is regarded as a reliable graph-theoretical-based analytic method with moderate to high test–retest reliability for the measurement of functional connectivity features of neural network nodes (Zuo and Xing, 2014). In graph theory, the DC of a node is defined as the number of functional connections to other nodes in the brain atlas (Bullmore and Sporns, 2009; Tomasi and Volkow, 2010). Thus, DC value could reflect functional connectivity features. Nodes with a higher DC are considered more important in the brain network, i.e., neural network hubs. Thus, DC results could indicate the highly connected regions of the brain and quantify the importance of each node in the brain network (Lv et al., 2018). DC features simplicity in understanding and performance. It has been successfully used to measure altered functional connectivity in idiopathic tinnitus (Chen et al., 2016), pulsatile tinnitus (Lv et al., 2015), schizophrenia (Zhuo et al., 2014), and Alzheimer’s disease (Dai et al., 2015). Alteration of DC in brain regions is closely correlated with symptom severity. In addition, DC at baseline could also provide objective neuroimaging-based indicators to predict response to treatment, such as in obsessive–compulsive disorder (Gottlich et al., 2015) and major depressive disorder (Shen et al., 2015). These results suggested the potential use of neuroimaging-based network features to predict response to treatment in tinnitus.

We hypothesized that functional connectivity features of the brain nodes determined by DC can predict the response of sound therapy through adjusted narrow band noise. In this study, we will analyze the functional connectivity of the neural network nodes within a standardized brain atlas (Power et al., 2011). We will compare the baseline and post-treatment functional brain network architectures in the cohort of patients with tinnitus. Furthermore, multiple regression analyses were applied to explore the potential of baseline functional connectivity features of neural network hubs to classify improvement status for patients.

## Materials and Methods

### Standard Protocol Approvals, Registrations, and Patient Consents

This experiment was approved by the Institutional Review Board (IRB) of Beijing Friendship Hospital, Capital Medical University, Beijing, China. Written informed consent was obtained from all subjects enrolled in this study. The protocol was registered on ClinicalTrials.gov, ID: NCT02774122.

### Participants

A total of 29 patients with untreated persistent tinnitus were recruited in this study. Twenty-eight age-, sex-, and education-level-matched healthy controls were also enrolled. The inclusion criteria for patients were as follows: aged 18 to 65 years and suffered from idiopathic tinnitus for more than 6 months persistently. Tinnitus was present as a hissing sound without any rhythm. Based on audiogram results, we excluded subjects with hearing loss, which was defined as more than 25 dB hearing loss at frequencies ranging from 250 to 8 kHz in puretone audiometry examination (PTA). Other exclusion criteria were hyperacusis on physical examination, otosclerosis, sudden deafness, Ménière’s disease, stroke, trauma, brain tumor, and other neurological diseases. The hearing thresholds of normal controls were also examined by PTA. Importantly, subject with hearing loss was one of the exclusion criteria. Other exclusion criteria for healthy controls were the same as above.

We asked patients to fill in the Tinnitus Handicap Inventory (THI) to assess the severity of disease at baseline (Newman et al., 1996). Tinnitus patients were treated by sound therapy through adjusted narrow band noise for 12 weeks, 20 min each time for three times per day. The intervention sound was matched with the clinical examination results, including loudness, and pitch matching. Specifically, the initial volume of noise sound was set as 5 dB over the loudness of tinnitus. For the frequency of the intervention sound, we first determined the tinnitus frequency (Tf) according to the results of tinnitus pitch matching. We then set an adjusted narrow band noise 1 kHz narrow band, Tf frequency. It would be necessary to adjust treated sound parameters based on examination every 2 weeks if the tinnitus sound is changed following treatment. We also asked the patients to fill out the THI again at the end of treatment. A reduction of at least 16 points in THI was considered effective (Zeman et al., 2011). Two of the patients were excluded due to lack of response to treatment according to this criterion.

### MRI Data Acquisition

For both tinnitus patients and healthy controls, MRI data acquisition was performed at baseline. All of the subjects were scanned using a 3.0 T GE (General Electric) scanner with an eight-channel head coil. Resting-state functional magnetic resonance imaging (fMRI) data were obtained with the following parameters (echo planar imaging sequence): TR/TE = 2,000/35 ms; field of view (FOV) = 240 × 240 mm; flip angle = 90°; matrix = 64 × 64; 28 slices; 4-mm slice thickness; 1-mm gap; and 200 time points in total. Additionally, high-resolution structural images were obtained with the following parameters: TR/TE/TI = 8.8/3.5/450 ms; FOV = 240 × 240 mm; flip angle = 15°; matrix = 256 × 256; 196 slices; and 1 mm thickness without gap.

### Image Preprocessing

Image preprocessing was performed using SPM 8^1^ and the Graph-theoretical Network Analysis Toolkit v2.0.0 (GRETNA^2^) in MATLAB (Wang et al., 2015). Our procedures included discarding the first 10 time points, slice timing, realigning, correcting head motion, spatial normalization to the MNI (Montreal Neurological Institute) space with resampled images (3 mm × 3 mm × 3 mm), detrending, and bandpass filtering (0.01–0.08 Hz). Nuisance variables including signals of white matter and ventricular and friston 24 head motion parameters (Yan et al., 2013) were regressed out. For the quality control, one of the enrolled healthy controls was excluded according to the exclusion criteria (spatial movement in any direction more than 2.0 mm or degrees). Notably, we did not perform smoothing in the image preprocessing procedure to prevent the possible introduction of artifactual correlations in the next DC calculation step (Zuo et al., 2012; Lv et al., 2018).

### Degree Centrality Calculation and Network Construction

Qualified images were subjected to further brain network construction and DC calculation using the GRETNA toolbox (Wang et al., 2015). A total of 264 defined brain nodes covering the whole brain were set as invested regions of interest (ROIs), as described in a widely used standardized brain atlas (Power et al., 2011). There were two main approaches when calculating DC: binary graph and weighted graph. The binary graph of DC is calculated as the number of functional connections. The weighted graph is computed as the sum of weights over every functional connection, providing a more precise centrality characterization of brain networks (Cole et al., 2010). Weighted networks generate more reliable numerical results compared to binarized networks (Wang et al., 2011). Thus, we adopted a weighted graph to calculate DC in this study. Specifically, the functional connectivity among ROIs was measured by computing the Pearson correlation coefficients (*r*) among the time series of every possible pair nodes. After calculation, all of the resulting DC maps were spatially smoothed (a Gaussian kernel with a full-width at half maximum = 6 mm). Finally, the DC maps were *z*-transformed (*z*-values of DC) to allow for averaging and between-subject comparisons.

### Statistical Analysis

#### Sample Characteristics and Degree Centrality Differences Among Groups at Baseline

The demographic data of the patients and healthy controls were compared using SPSS 12.0 software (SPSS, Inc., Chicago, IL, United States) through two-sample *t*-tests, paired two-sample *t*-tests, and a Fisher’s exact test. The results were considered significant with *P* < 0.05. Longitudinal changes in THI scores were calculated as: ΔTHI = THI_pre_ - THI_post_. The ΔTHI would represent the therapeutic effect of treatment. Imaging data were analyzed in the SPM. Differences in the *z*-values of DC between tinnitus patients and healthy controls were analyzed by a two-sample *t*-test (false discovery rate (FDR) corrected for multiple comparisons, *P* < 0.05). The results were visualized with the REST Slice Viewer and BrainNet Viewer^3^ (Song et al., 2011; Xia et al., 2013).

#### Degree Centrality Differences at Baseline

After DC calculation and network construction, we produced a continuously weighted network (264 × 264 matrix) for each subject. To reveal the differences of DC between tinnitus patients and healthy controls at baseline, we used a two-sample *t*-test. The results were FDR corrected with *P* < 0.05.

#### Correlation Between Degree Centrality Maps and Tinnitus Handicap Inventory Score

According to a one-sample Kolmogorov–Smirnov test, the THI score at baseline and the ΔTHI values were both normally distributed. To identify brain nodes that were significantly related to the outcome of treatment for tinnitus patients, we conducted a correlation analysis between the *z*-values of DC and baseline THI as well as ΔTHI. Age and gender were added as covariates during the calculations.

#### Adjusted Receiver Operating Characteristic Curve

To further examine whether the DC at baseline might be sufficiently sensitive and specific to serve as a potential neural biomarker for predicting improvement after intervention, we employed adjusted receiver operating characteristic (ROC) curves (Janes et al., 2009). We added age, gender, and education as covariates to recalculate ROC results. Analytic strategies were performed for each node whose baseline DC was significantly correlated with the clinical response of treatment in tinnitus patients.

Additionally, according to the standard defined by Zeman et al. (2011), a reduction of at least 40 points in THI represents relative better effects of the therapy. Thus, we set THI = 40 as a cutoff value to identify brain regions that predict improvements after treatment with high specificity and sensitivity.

## Results

### Demographics and Clinical Characteristics

A total of 27 tinnitus patients and 27 healthy controls completed the study (Table 1). For tinnitus patients, THI scores longitudinally decreased significantly. ΔTHI were also calculated.

**Table 1:** Summarized demographic and clinical characteristics of the enrolled subjects.

	Tinnitus patients (baseline, *n* = 27)	Tinnitus patients (12th week, *n* = 27)	Healthy controls (baseline, *n* = 27)	Healthy controls (12th week, *n* = 27)	*P*-value
Age (years)	23–62 (37.5 ± 10.6)		25–59 (38.4 ± 11.3)		0.78^a^
Gender (male/female)	12/15		12/15		> 0.99^b^
Education (years)	6–16 (11.4 ± 3.0)		6–19 (12.3 ± 3.6)		0.33^a^
Handedness	27 right-handed		27 right-handed		> 0.99^a^
Tinnitus duration (months)	6–48 (25.0 ± 13.4)		NA		NA
THI score	40–90 (66.6 ± 13.9)	12–50 (30.4 ± 11.1)	NA		< 0.01^c^
ΔTHI score	24–52 (36.2 ± 8.8)		NA		NA

*Data are presented as min–max ranges (mean ± SD) for all variables except gender. THI: Tinnitus Handicap Inventory. ΔTHI = THI_pre_ - THI_post_. NA: not applicable. ^a^Two-sample t-tests. ^b^Chi-square test. ^c^Paired-sample t-tests.*

### Functional Connectivity Differences Between Patients and Healthy Controls at Baseline

We calculated the DC of the 264 defined brain nodes within the whole brain. As shown in Table 2, when compared to healthy controls, the anterior cingulate cortex (ACC), right insula, bilateral superior frontal gyrus (SFG), right middle frontal gyrus (MFG), right inferior parietal lobule (IPL), bilateral thalami, and right middle temporal gyrus [two separate brain regions: the anterior part overlapped within the default mode network (DMN) and the posterior part overlapped with the associate auditory cortex (AAC)] showed increased DC in tinnitus patients. No node with decreased DC was found. These results suggest that an objective neuroimaging-based indicator can differentiate tinnitus from healthy controls.

**Table 2:** Brain nodes with significant differences in DC values in tinnitus patients relative to healthy controls at baseline.

Brain region	Peak MNI, mm			DC value of tinnitus patients	DC value of healthy controls	*T*
	*x*	*y*	*z*			
Anterior cingulate cortex	−3	26	44	73.75 ± 3.21.25	57.42 ± 7.22.45	2.75
R insula	37	32	−2	70.87 ± 0.20.96	54.27 ± 4.18.42	3.09
L superior frontal gyrus	−20	45	39	73.75 ± 3.21.25	57.42 ± 7.22.45	2.75
R superior frontal gyrus	26	50	27	73.75 ± 3.22.17	57.50 ± 7.15.56	3.12
R middle frontal gyrus	43	49	−2	65.73 ± 5.24.29	48.06 ± 8.15.05	3.21
R inferior parietal lobule	49	−42	45	69.96 ± 9.23.02	52.69 ± 2.22.68	2.78
L thalamus	−15	4	8	84.88 ± 4.25.69	61.31 ± 1.21.43	3.66
R thalamus	9	−4	6	82.12 ± 2.27.21	61.84 ± 1.27.48	2.73
R middle temporal gyrus (anterior part, DMN)	65	−24	−19	74.87 ± 4.21.47	58.78 ± 8.21.19	2.77
L middle temporal gyrus (posterior part, SAC)	−56	−50	10	84.42 ± 4.25.85	66.14 ± 6.23.97	2.69

*For the DC values, data are presented as the mean ± SD. DC, degree centrality (note that the DC value was z-transformed); R, right; L, left; MNI, Montreal Neurological Institute; DMN, default mode network. SAC, secondary auditory cortex. Peak T scores and location of node maxima in MNI coordinates are listed. The significance threshold was set as P < 0.05 corrected for multiple comparisons using FDR.*

### Correlation Between Degree Centrality Maps and Tinnitus Handicap Inventory Score

**FIGURE 1:**
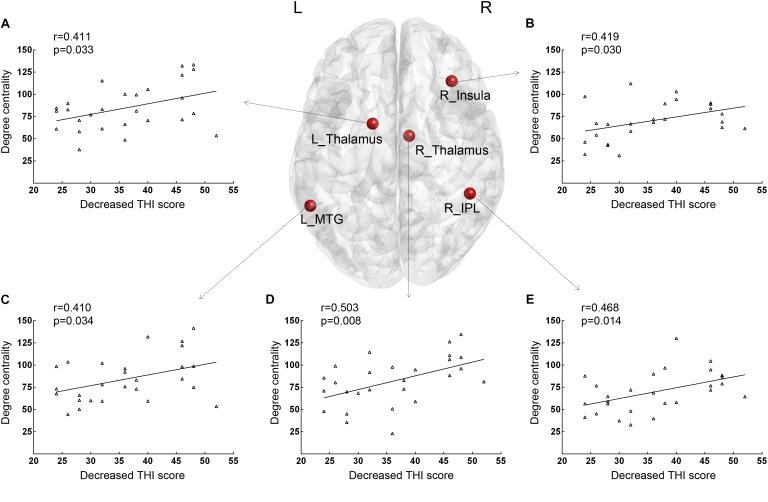
Brain nodes with baseline DC values that were significantly correlated with decreased THI scores after 12 weeks of sound therapy through adjusted narrow band noise. **(A)** Results of the left thalamus. **(B)** Results of the right insula. **(C)** Results of the left middle temporal gyrus. **(D)** Results of the right thalamus. **(E)** Results of the right inferior parietal lobule. L, left; R, right; DC, degree centrality; THI, tinnitus handicap inventory; MTG, middle temporal gyrus; IPL, inferior parietal lobule. X-axis, Decreased THI score; Y-axis, number of Degree Centrality.

Positive correlations between THI at baseline and DC values were found in the right insula (*r* = 0.415, *p* = 0.031). As shown in Figure 1, for correlative analysis of DC values and therapeutic effect, significant positive correlations between increased *z*-values of DC and ΔTHI were found in the right insula (*r* = 0.419, *p* = 0.030), right IPL (*r* = 0.468, *p* = 0.014), bilateral thalamus (left: *r* = 0.411, *p* = 0.033; right: *r* = 0.503, *p* = 0.008, respectively), and posterior part of the left MTG (SAC; *r* = 0.410, *p* = 0.034) after age and gender correction. There were no additional significant correlations reported in this study.

### Sensitivity and Specificity of Baseline Degree Centrality in Predicting Treatment Effect

**FIGURE 2:**
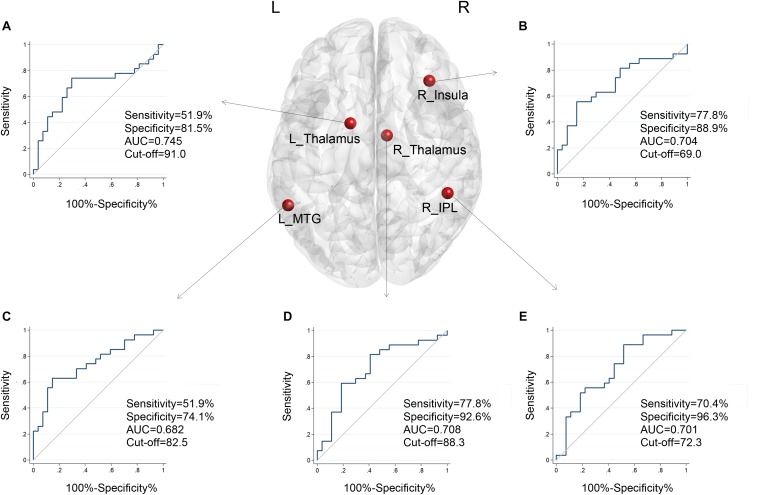
The adjusted ROC curves and AUC values of the five nodes when classifying improvements in tinnitus patients after treatment, with age, gender, and education as corrections. **(A)** Results of the left thalamus. **(B)** Results of the right insula. **(C)** Results of the left middle temporal gyrus. **(D)** Results of the right thalamus. **(E)** Results of the right inferior parietal lobule. X-axis, 100%-Specificity%; Y-axis, Sensitivity.

According to the standard, a reduction of at least 40 points represents better effects of the therapy (Zeman et al., 2011). We further tested whether the DC had predictive value for treatment effectiveness of the intervention. The ROC analyses revealed that these five brain regions performed well in classifying better effects of therapy after 12 weeks of treatment. As shown in Figure 2, after age, gender, and education correction, the adjusted area under the curve (AUC) values of the nodes were recalculated. The functional connectivity features of other brain nodes were as follows: right insula, AUC = 0.704, sensitivity = 77.8%, specificity = 88.9%, cutoff value = 69.0; right IPL, AUC = 0.701, sensitivity = 70.4%, specificity = 96.3%, cutoff value = 72.3; left thalamus, AUC = 0.745, sensitivity = 51.9%, specificity = 81.5%, cutoff value = 91.0; right thalamus, AUC = 0.708, sensitivity = 77.8%, specificity = 92.6%, cutoff value = 88.3; and posterior part of left MTG (SAC), AUC = 0.682, sensitivity = 51.9%, specificity = 74.1%, cutoff value = 82.5. We noted that the AUC of bilateral thalami had the highest values (left, 0.745; right, 0.708). The observed AUC values and bias are listed in Table 3. We also added unadjusted AUC values of each node to illustrate the impact of covariates on ROC values in tinnitus research.

**Table 3:** The adjusted AUC values and bias of the brain nodes.

	AUC (unadjusted; adjusted)	Bias	Bootstrap standard error	95% confidence interval (unadjusted; adjusted)
R insula	0.713			0.574, 0.828
	0.704	0.00819	0.07431	0.539, 0.831
R inferior parietal lobule	0.716			0.577, 0.830
	0.701	−0.00043	0.08184	0.524, 0.844
L thalamus	0.752			0.615, 0.859
	0.745	0.00027	0.07514	0.575, 0.868
R thalamus	0.704			0.564, 0.820
	0.708	0.00740	0.07941	0.535, 0.846
L middle temporal gyrus (posterior part, SAC)	0.700			0.560, 0.817
	0.682	−0.00336	0.08011	0.528, 0.835

*95% confidence interval: bias-corrected confidence interval. R, right; L, left; SAC, secondary auditory cortex.*

## Discussion

This study demonstrated the potential of using a neuroimaging biomarker in predicting the therapeutic improvement of tinnitus patients. To our knowledge, this is the first study to evaluate the predictive value of the graph-theoretical metric parameters for the outcomes of sound therapy through adjusted narrow band noise.

Based on a widely used standardized brain atlas consisting of 264 brain nodes, we first identified 10 brain nodes with significant differences in their DC values between tinnitus patients and healthy controls. These brain nodes were widely distributed within the whole brain and were mainly located in the frontal lobe (left SFG, right SFG and MFG), parietal lobe (right IPL), and ACC. These brain regions largely overlap with the emotional processing areas, which are core structures within the fronto-parietal-cingulate network (Golm et al., 2013; Husain, 2016). Additionally, the thalamus, identified as an important brain node within the thalamocortical pathway in mediating auditory–limbic interactions, had a significantly increased DC in tinnitus patients compared to healthy controls at baseline. After retrospective data analysis, we identified five nodes with significant correlations between their DC values at baseline and decreased THI scores after treatment. Importantly, the DC of these five nodes had different predictive value for better treatment responses. Among which, the left thalamus was one of two brain regions with the highest AUC values, while the other brain region is the right IPL. However, after age, gender, and education correction, the adjusted AUC values for the bilateral thalami were the highest (left, 0.745; right, 0.708). According to our results, the DC value of the thalamus is an objective neuroimaging-based indicator to predict better response to treatment. An improved understanding of neural correlates of tinnitus after therapy should motivate the development of innovative neural-based means for assessing the efficacy of sound therapy through adjusted narrow band noise.

### Methodological Considerations

When analyzing functional connectivity features of the neural network, there are several methods that can be applied. Seed-based functional connectivity is one of the most commonly used methods. The coupling of time series between brain areas indicates that they are involved in the same underlying functional process, and thus can be interpreted as functionally connected (Lv et al., 2018). The computation is relatively simple, and the results are easily understood. The modest to high test–retest reliability across connections (Shehzad et al., 2009) further facilitates its application. However, one of the major disadvantages of seed analysis is its dependence on the selection of ROIs in conducting ROI-wise analysis. When the selected seed changes, it may limit the reproducibility of the results. Thus, it would be more precise if selected brain nodes are defined prior to data analysis and set as a standard atlas.

To overcome the problems mentioned above, we first applied the DC analytic method. Featuring high test–retest reliability (Zuo et al., 2012), the DC values reflect the entirety of functional connections between one node to other nodes in the brain atlas, rather than identifying each connection (Bullmore and Sporns, 2009; Tomasi and Volkow, 2010). Thus, the results of DC are quantified values that characterize the importance of a node (Lv et al., 2018). For the definition of nodes, we applied a widely used standardized brain atlas, which included 264 defined nodes (Power et al., 2011). Thus, neural activity could be measured among those 264 predefined nodes within a graph theoretic framework without selection bias. These methodological considerations increased the validity of results and minimized possible confounding factors in this study.

Results of correlation analysis were also needed to be discussed. We performed correlative analysis with both baseline THI score at baseline and the ΔTHI. However, for the results, we only found positive correlations between the DC values and THI score at baseline in the right insula, but there were five brain nodes whose DC values were positively correlated with the ΔTHI, including the right insula. It indicated that correlations between brain activity and THI score or ΔTHI score may be different. One reason may be the different focused aspect when we do statistical analysis. One focused on the cross-sectional aspect and the other focused on the longitudinal changes. As a result, results may be different and not necessarily overlapped. Similar phenomena also exist in a previous study on patients with obsessive–compulsive disorder (Gottlich et al., 2015). Thus, it is reasonable to presume that results may be different and not be necessarily overlapped. For another reason, it may indicate that the DC value is more appropriate for prediction of treatment effect longitudinally rather than assessment of disease severity. It would be also one of the advantages that we apply DC as a predictive biomarker in this study.

### The Involvement of the Fronto-Parietal- Cingulate Network in Tinnitus

The fronto-parietal-cingulate network has been implicated in tinnitus (Golm et al., 2013; Husain and Schmidt, 2014; Husain, 2016). Brain regions related to the fronto-parietal-limbic network may include the anterior and PCC, insula, frontal brain areas (SFG, MFG, especially dorsal lateral prefrontal cortex), amygdala, parahippocampal gyrus (limbic), and precuneus. These brain regions are closely correlated with emotional processing. This network is more active in patients with highly distressing tinnitus and high THI scores and is also a specific distress network in tinnitus (Golm et al., 2013). In another study, increased functional connectivity was detected between the frontoparietal regions and other parts of the brain (Maudoux et al., 2012). In the current study, we also found increased functional connectivity of several regions within the fronto-parietal-cingulate network, which was reflected by increased DC values. As a result, our study provided additional evidence that FC of this network was mainly increased in the perception of tinnitus. The functional alterations of the fronto-parietal-cingulate network as well as anatomical changes were involved in the perception of tinnitus. Gray matter volume changes in the thalamic level were considered to be critical findings of tinnitus (Muhlau et al., 2006). According to recent studies, correlations of tinnitus distress with cortical thickness and cortical surface area in bilateral cingulate cortex were also reported (Meyer et al., 2016). Morphological changes were also detected in frontal areas after treatment (Krick et al., 2015) and were interpreted as tinnitus-specific gray matter alterations, which was closely correlated with emotional changes of tinnitus. Additionally, the THI score of the patients was 66.6 ± 13.9 at baseline, representing a relatively high distress state. Thus, it is reasonable that core structures of the fronto-parietal-limbic network with significantly increased functional connectivity could be identified. Our study further supports the involvement of this neural network in tinnitus processing.

The THI score also significantly decreased after treatment in this study, representing alleviation of symptoms. However, we did not identify correlations between decreased THI scores and the DC values of most regions of the fronto-parietal-limbic network at baseline, except for the right insula. The nature of THI may be one reason. In fact, THI is a moderately accurate test for assessing psychiatric disorder of tinnitus patients (AUC = 0.792, according to previous study) (Salviati et al., 2013). As one of the most widespread validated questionnaires in the tinnitus research field (Newman et al., 1996), it is commonly applied to quantify tinnitus severity rather than tinnitus-related distress (Langguth et al., 2007). For the evaluation of distress, both the Global Severity Index (GSI) of Symptomatic Check List-90-Revised (SCL-90-R) and Stress-related Vulnerability Scale (VRS) have higher sensitivity and specificity (Salviati et al., 2013). Thus, a decrease in the THI score may not be specific to relief from tinnitus-related distress. It would be helpful to acquire GSI or VRS scores to specifically investigate the activity of the fronto-parietal-limbic network.

For the right insula, we identified positive correlations between the DC value and baseline THI as well as a decreased THI score. It may represent that functional connectivity of the right insula would be an indication for severe patients and may also indicate that high functional connectivity of the right insula have potential predictive value for effective treatment. The insula is one of the major hubs that modulate tinnitus-related distress (Husain, 2016). As a core structure of the salience network, many studies have indicated the important role of the insula in emotional processing, as reviewed by De Ridder et al. (2014). When it is active, an external auditory stimulus is perceived by the subject (Sadaghiani et al., 2009). For tinnitus patients, an elevated response of functional activity was found in the right insula and bilateral parahippocampus when processing affective stimuli (Carpenter-Thompson et al., 2014). The insula and ACC are more active in chronic tinnitus compared to recent-onset tinnitus (Vanneste et al., 2011). Greater functional connectivity was also detected in both the right anterior insula and left inferior frontal gyrus in a tinnitus group compared with healthy controls (Burton et al., 2012). Abnormal regional neural activity and functional interactions between the right insula and DMN and auditory network were also reported in patients with persistent pulsatile tinnitus (Lv et al., 2017). Anatomical changes were also reported in the insula, according to a voxel-based morphometry study with a large sample (Schecklmann et al., 2013). Those studies indicated the importance of the insula in tinnitus mediation. In this study, we found that the insula DC value performed well in classifying improvements of the tinnitus patients after treatment. Our study provided additional evidence for this core structure in the salience network in tinnitus.

### Auditory–Limbic Interaction Pathway Through the Thalamus

The thalamus is the subcortical auditory processing region. The medial geniculate nucleus in the thalamus, accompanied by the primary and secondary sites in the superior temporal gyrus, forms a typical auditory perception pathway. Further, thalamic function plays a critical role in the perception of tinnitus and in regulating the flow of signal among the auditory network and limbic network. One hypothesis concerning tinnitus perception is that the thalamic reticular nucleus mediates information between the limbic and auditory cortex. Under normal circumstances, the unwanted signal is inhibited in the thalamus, and thus the propagation of tinnitus is reduced. In chronic tinnitus, cortico-thalamic activity is increased and prevents inhibition of the tinnitus signal (Rauschecker et al., 2010; Zhang, 2013). Thus, changes in the cortico-thalamic feedback loop result in increased alteration of functional connection of the thalamus (Leaver et al., 2011). In our study, we mainly enrolled patients with moderate to severe tinnitus. Those patients were also featured by relatively higher DC value of the bilateral thalamus. Although we did not find a positive correlation between DC value and THI score at baseline, increased FC of the bilateral thalamus could also further support this theory.

In a different model, the thalamus also plays a major role in the development of tinnitus. As analyzed by magnetoencephalography (MEG), the thalamo-cortical loop has normal rhythms in the normal state relating to sleep and consciousness (Llinas and Ribary, 1993). When there is reduced functional input to the thalamus or protracted functioning of the thalamus, the rhythms of the thalamus change to a large-scale but slow-rate oscillatory coherent activity, reducing lateral inhibition and disinhibiting high-frequency gamma waves (Llinas et al., 1999). To compare the results, the relationship between fMRI and MEG was preliminarily analyzed (Houck et al., 2017). The spatial patterns of the two kinds of analysis were similar but not identical. Interestingly, the functional connectivity results may be inversed in the two kinds of studies. Thus, theoretically, the thalamus would be identified with increased FC in fMRI analysis, as demonstrated by increased DC in our study. As a result, for both of the models, it was reasonable that thalamus plays a critical role in mediating tinnitus perception. Besides, similar to a previous study, our results may further indicate the inverse relationship of FC revealed by MEG and fMRI analyses.

To further validate the proposed tinnitus models, one previous study specifically analyzed the functional connectivity features of the thalamus in tinnitus patients (Zhang et al., 2015). Both increased and decreased FC were detected between the left and right thalamus and brain regions in the cerebral cortex. Different from the results of this study, we only identified increased functional connectivity (indicated by higher DC values) in the bilateral thalamus relative to healthy controls. There are two possible reasons. Firstly, similar to the introduction and methodological considerations mentioned above, DC calculates the sum of weights over every functional connection (Cole et al., 2010), while functional connectivity measures the connection between several brain regions. Thus, the connectivity feature of each brain node revealed by DC values will be averaged. There might be decreased FC between the left or right thalamus and other brain nodes, but this effect may be covered up by increased FC values in this study. This circumstance may also explain why the sensitivity and specificity of the DC values of both the left and the right thalamus were only moderate to high in predicting the therapeutic effect. This is also one limitation of the DC analytic method. However, we should also pay special attention to the accuracy of traditional ROC. As suggested by Holly Janes, covariates, e.g., age and gender, are factors that should be incorporated into ROC analysis (Janes et al., 2009). Thus, in this study, we added age, gender, and education as covariates to recalculate the ROC results. After correction, the adjusted AUC values for the bilateral thalami were the highest (left, 0.745; right, 0.708). These results further highlighted the importance of the thalamo-cortical pathway in processing tinnitus information.

Secondly, a previous study (Zhang et al., 2015) identified disconnection of the FC of the right thalamus and left STG, especially in patients with extended disease duration (∼10 years). Additionally, decreased FC was negatively correlated with tinnitus duration, possibly indicating habituation of tinnitus. In our study, the disease duration of enrolled tinnitus patients was less than 48 months. As a result, the disconnection effect may not be obvious in patients in the early stage of disease, resulting in a relatively high DC value at baseline. Sound therapy also has habituation effects on tinnitus. In our research, higher DC values in the bilateral thalamus are considered to be potential biomarkers for predicting the effect of sound therapy through adjusted narrow band noise. Thus, in light of the previous study, we hypothesize that DC of the thalamus decreases after treatment. As this is a core structure that mediates information between the limbic and auditory cortex, it is critical to test our hypothesis by analyzing the functional connectivity features of the thalamus in patients with longer disease duration, as well as the alteration of DC value of bilateral thalamus after sound therapy in further studies.

### Limitations

There are several limitations of this study. Firstly, we excluded two patients who did not respond to treatment in this study. However, only two subjects would not be enough to do statistical analysis in an fMRI study. It is important to analyze brain functional activity in these kinds of patients in order to provide more information about the neural mechanism of sound therapy and help to improve treatment strategy in further studies. Secondly, tinnitus patients enrolled in the current study did not show any degree of hearing loss, which is not representative for most tinnitus patients. Thirdly, it would be much more helpful to enroll a tinnitus group with sham treatment in order to exclude the placebo effect of tinnitus treatment. Last but not least, we applied adjusted narrow band noise (1 kHz narrow band, Tf ± 0.5 kHz) for the treatments of patients, but it indicated that we were providing more energy in high frequencies where critical bands are narrower. It would be better if octave bands or similar bands were applied so that critical bandwidth was considered in further studies.

## Conclusion

Our study further supports the involvement of the fronto-parietal-cingulate network in mediating tinnitus and suggests that the DC value of the thalamus at baseline is an objective neuroimaging-based indicator for predicting effectiveness of sound therapy through adjusted narrow band noise. This preliminary analysis has important implications in motivating innovative neuroimaging-based approaches for predicting the efficacy of treatment, thus guiding earlier personalized therapeutic strategies for tinnitus patients. Further studies analyzing functional connectivity of the thalamus are still needed to further validate the predictive effect of its DC value at baseline.

## Ethics Statement

This study was carried out in accordance with the recommendations of LH of guidelines, the Institutional Review Board (IRB) of Beijing Friendship Hospital, Capital Medical University with written informed consent from all subjects. All subjects gave written informed consent in accordance with the Declaration of Helsinki. The protocol was approved by the Institutional Review Board (IRB) of Beijing Friendship Hospital, Capital Medical University.

## Author Contributions

LH designed the experiments, collected the data, performed the statistical analysis, and wrote the manuscript. ZN conducted the statistical analysis. LC and ZoP contributed to the discussion and manuscript revision. LC, WH, CX, WZheng, and ZgP collected the data. WZhenchang, YZ, and GS are guarantors of this work. LH and WZhenchang are the corresponding authors of this manuscript, who have full access to all the data in the study and take responsibility for the integrity of the data and the accuracy of the data analysis.

## Conflict of Interest Statement

The authors declare that the research was conducted in the absence of any commercial or financial relationships that could be construed as a potential conflict of interest.

## Footnotes

1 http://www.fil.ion.ucl.ac.uk/spm

2 http://www.nitrc.org/projects/gretna/

3 http://www.nitrc.org/projects/bnv/
